# Data Safe Havens and Trust: Toward a Common Understanding of Trusted Research Platforms for Governing Secure and Ethical Health Research

**DOI:** 10.2196/medinform.5571

**Published:** 2016-06-21

**Authors:** Nathan Christopher Lea, Jacqueline Nicholls, Christine Dobbs, Nayha Sethi, James Cunningham, John Ainsworth, Martin Heaven, Trevor Peacock, Anthony Peacock, Kerina Jones, Graeme Laurie, Dipak Kalra

**Affiliations:** ^1^ Institute of Health Informatics University College London London United Kingdom; ^2^ Institute of Health Informatics and Institute for Women's Health University College London London United Kingdom; ^3^ Farr Institute CIPHER Swansea University Swansea United Kingdom; ^4^ Farr Institute Scotland School of Law University of Edinburgh Edinburgh United Kingdom; ^5^ Mason Institute School of Law University of Edinburgh Edinburgh United Kingdom; ^6^ Farr Institute University of Manchester Manchester United Kingdom; ^7^ IT for SLMS Information Services Division University College London London United Kingdom; ^8^ EuroRec: The European Institute for Health Records Brussels Belgium

**Keywords:** trusted research platforms, data safe havens, trusted researchers, legislative and regulatory compliance, public engagement, public involvement, clinical research support, health record linkage supported research, genomics research support

## Abstract

In parallel with the advances in big data-driven clinical research, the data safe haven concept has evolved over the last decade. It has led to the development of a framework to support the secure handling of health care information used for clinical research that balances compliance with legal and regulatory controls and ethical requirements while engaging with the public as a partner in its governance. We describe the evolution of 4 separately developed clinical research platforms into services throughout the United Kingdom-wide Farr Institute and their common deployment features in practice. The Farr Institute is a case study from which we propose a common definition of data safe havens as trusted platforms for clinical academic research. We use this common definition to discuss the challenges and dilemmas faced by the clinical academic research community, to help promote a consistent understanding of them and how they might best be handled in practice. We conclude by questioning whether the common definition represents a safe and trustworthy model for conducting clinical research that can stand the test of time and ongoing technical advances while paying heed to evolving public and professional concerns.

## Introduction

The challenges of secure electronic health care records reuse and its trustworthiness are well recognized [1]. The international clinical research community is nevertheless continually recognizing the significance of big data for driving research and deriving further benefit for patient care and outcomes [2,3]. While these challenges remain internationally applicable, we focus in this paper on the recent experiences across the United Kingdom to illustrate an ongoing dilemma and challenges around the sharing and wider linkage of health and social care records encouraged by the big data trend, and how established protection strategies must continue to evolve to meet them.

In considering the ongoing dilemma, we discuss the paradigm of the data safe haven (DSH) that has garnered increasing interest across the UK research community. This paradigm is a commonly recognized, state-of-the-art approach for handling information derived from health care records in clinical research, which has also achieved international recognition. While the paradigm has developed to include a set of 12 criteria, including the need to take account of societal concerns and anxieties when handling data within any environment that claims to be a safe haven [4], there remains work to be done to develop a more inclusive definition of trustworthiness in this context, specifically with regard to the public and its views on security [5]. But what does the paradigm look like in practice and how does it measure up against developing dilemmas and challenges in the age of big data? We aim in this paper to answer this question by discussing the practical experience of establishing and running DSHs. With reference to a series of case studies across the 4 nodes of the Farr Institute of Health Informatics Research, which spans the United Kingdom, we build upon the understanding that has developed around the DSH paradigm and the need to apply a more developed and inclusive understanding of trust as it applies to different stakeholders.

We use the case studies to identify comparable features of the 4 nodes as they have developed and evolved independently. Using this and a detailed consideration of the legal, regulatory, and information security requirements, we examine the ramifications of their implementation in practice for clinical research with regard to the established criteria. This provides a basis to recommend an approach for fostering and nurturing trust across stakeholders as the linkage trends and dilemmas continue to evolve. We argue that the development of such trust relies on the engagement with and involvement of the public in the requisite governance and oversight of any system if it is to be trusted. We emphasize that, in practical terms, the DSH paradigm crucially must recognize that the management of risk and support of trustworthy, careful working practice is not a feature provided solely by encryption and access control solutions, the physical security of data centers, or the control of dataset release, but also by effective training, education, and accreditation of the people using those systems so that they understand how best they can work safely and securely, in compliance with legal, regulatory, and ethical requirements. While the focus of the work has been on the UK experience, the discussion is intended to inform the identified challenges of electronic health records reuse internationally.

## The Big Data Dilemma

Big data in practice involves linking information from electronic health care records with records contained in disease registries and data generated by genome sequencing initiatives such as the 100,000 Genomes Project [6] or the Electronic Medical Records and Genomics Network [7]. The potential to link with data collected from social care services has also been identified as a key theme for research strategy [8], and there is governmental support for both in terms of funding [9] and legislative focus, for example, to aid health and social care policy development [10].

This trend has been controversial, and anxieties about upholding the medical profession’s duty of confidence to their patients, protecting the patient’s right to a private life, and compliance with data protection legislation have continued to emerge. Studies that have explored attitudes toward using health and other social care records for research point to general support for research uses [11], which may, however, be conditional on obtaining consent [12]. This must be taken in the context of an identified “data trust deficit,” where the UK Royal Statistical Society has found that people trust organizations’ (such as the UK National Health Service, NHS) uses of data less than the organizations themselves [13]. There have also been public anxieties over the handling of initiatives such as the care.data program in England [14] and more recently proposed initiatives in Scotland [15]. Some concerns have been expressed about the use of health record information for profit by industry [16], and there is evidence to suggest that legal and regulatory compliance may not be enough to win wider public and professional support for all of the intended uses of information captured during health care [17].

This apparent dilemma is compounded when viewed both from the research—especially from the epidemiological—perspective, where there is evidence that gaining explicit consent using opt-in from participants reduces population sample sizes significantly and can introduce selection bias [18-23], and from a realist perspective, where gathering consent is not always possible or rules out a firm basis on which to process data [22,24,25]. This must be coupled with discoveries that research participants are expecting greater transparency about [26] and a “louder voice” in how research is conducted [27]. The dilemma is clearly one that straddles both ethical and legal requirements and requires balancing the rights of the individual—particularly around autonomy—and the rights of the wider citizenry to benefit from scientific progress [5].

In addition to this, and regardless of the measures taken to protect participants as guided by the law and research ethics, there remains some residual risk of harmful outcomes, particularly if participants are accidentally or with some effort deliberately re-identified within a research dataset. Methods to render records anonymous cannot guarantee anonymity [28-30], meaning that risks of participant re-identification, and therefore of harm, remain. These risks are becoming recognized as being more likely with genome research [27]. De-identification might, however, not always be the best approach to take: in 2006 the UK Academy of Medical Sciences identified in its report on using personal data in health research that meaningful research needed varying degrees of identifiable data because “...most important research using personal data requires access to identifiable data at some point for some purpose...” [31]. This issue has surfaced in practice, where de-identification is being used as a means to limit disclosure and protect the confidentiality of health care records at the expense of data utility for research [32] and is an impediment to research itself [33]. This is further illustrated when the risk of detrimental effects to data quality and efficiency is heightened if disclosure risk is handled in isolation. This is problematic in cases where analytic strength needs to be “borrowed” from one data source by another to realize its public benefit, where data being borrowed can be processed without needless re-identification provided its governance is not handled independently of the borrower dataset [34].

A balance therefore needs to be found between the extent of de-identification and the utility of data for research, which reemphasizes the importance of handling these risks according to legislated requirements and meaningfully supported, trusted, careful, and secure working practice that works at scale. But what does that entail in practice and, crucially, what extent is needed to protect participants and the research community, and also to meaningfully address public concerns while honoring the rights of the individual?

## What Is the Data Safe Haven Paradigm and Where Did It Come From?

The concept of the DSH pertaining to the United Kingdom has been developing since the early 1990s and continues to elude a rigid or specific definition [4]. The garnering of the DSH paradigm in the UK research community in particular is well illustrated by the 2008 Data Sharing Review [35], which emphasized the importance of handling health care data safely and securely for research purposes. It recommended the development of safe havens, which were identified as secure working environments that required levels of accreditation for researchers, as well as certification for data handling facilities that were in line with high standards of information security.

The more recent Information Governance Review in 2013, in which information-handling practices in England were extensively reviewed by an independent, Department of Health-appointed panel, has endorsed this recommendation [36]. It identified the importance of the safe haven paradigm and made further recommendations about levels of compliance with existing codes of practice. These included the Information Governance Toolkits across the UK jurisdictions, as well as independent certification of compliance with standards such as the International Organization for Standardization (ISO)/International Electrotechnical Commission 27001 standard on information security management [48]. The ISO standard establishes the requirements for information security management and helps to mold legal prescription into practical tools for use in working practice. ISO 27001 offers an opportunity for independent certification by ISO-accredited information security experts, which in turn provides higher levels of assurance around the security of certified systems.

In 2014, the Academy of Medical Sciences hosted a meeting about DSHs in research to better understand what had been developed and how they were working. The meeting identified a need for developing a common definition of the DSH in practice. Additionally, emphasis was placed on the importance of developing these DSHs with due regard to providing performance metrics and success criteria, research, training, and educational needs, as well as understanding public expectations by means of meaningful, ongoing engagement and potential involvement [37]. By reviewing the state-of-the-art in safe working practice for clinical research, the aim of the meeting was to bring a common understanding to the wealth of legislative, regulatory, and practical requirements that underpin information governance in clinical research practice.

Since the 2014 meeting, commentary and discussion around the understanding of the DSH paradigm have continued, and evidence has emerged that this is becoming an internationally recognized concept. Burton et al [4] have provided a set of 12 criteria to define the meaning of DSH. The criteria are focused on trustworthiness and reliability of the data that are provided, on upholding legal and ethical requirements, and on managing and releasing data within the bounds of social acceptability. The criteria also relate to maintaining the security of the data, specifically around the preservation of confidentiality, integrity, and availability of the data, and appropriate and secure access to identifying data and their protection [4]. Knoppers and Chadwick conclude that “[c]lear systems of governance, public trust in data security, personal empowerment and the responsibility it brings re ‘knowing’ (or not) as well as transparency of research outcomes are to be welcomed...” [5]. They have further developed an understanding of the ethics involved in this area and expanded the scope of “trustworthiness” to include the public and its views on the security of safe havens. In this paper, we consider these 12 criteria and the more inclusive scope defining trustworthiness with a deeper discussion of legal, ethical, and risk management requirements.

### Bases in Law for Information Governance in Research in the United Kingdom

We refer to the main acts of law and common law that are in place to govern health research and protect information as it is used for these purposes in the United Kingdom. We use the UK legislature to describe the bases in law because we will discuss implementations of the DSH paradigm in research platforms across three jurisdictions in the United Kingdom: Wales, Scotland, and England. To summarize, the bases in law stem from a focus on protection of individuals and the definition of professional duties with the common law duty of confidentiality and its variations across UK jurisdictions. There are also statutory provisions around consent for research and protections for vulnerable groups in the Children Act 1989 [38] and the Mental Capacity Act of 2005 [39], and for using biological samples for research in the Human Tissue Act of 2004 [40]. The legislature further recognizes the right to a private life in the Human Rights Act of 1998 [41]. The more data-focused Data Protection Act of 1998 [42] defines statutory requirements for handling data to protect the individuals about whom data have been recorded, compliance with which is overseen by an Information Commissioner who has powers to fine organizations for serious breaches. The Information Commissioner also oversees compliance with European regulations regarding electronic communications [43].

Further statutory provision exists in the form of the Health and Social Care Act of 2012 [44], which provides a basis in law for processing information to support health and social care services, as well as the Health and Social Care Information Centre in England, an organization responsible for handling health and social care information and for gathering large research datasets, which was originally identified as an accreditor of safe havens. The Care Act of 2014 [45] defines the need for ethical approval of health research via processes laid out by the Health Research Authority in England and Wales, and requires that the Health and Social Care Information Centre handle data with due regard to privacy. Additional support in England and Wales lies in Section 251 of the National Health Service Act of 2006 [46], which empowers the Secretary of State for Health to set aside the common law duty of confidentiality, where applicants must show regulatory compliance and show a substantial public interest for setting aside the common law, a power that in Scotland lies with Caldicott Guardians, senior figures who safeguard the confidentiality of patient data in the NHS and enable appropriate information sharing. While this armory of legal protections enforces the requirement of careful working practice and processing that should not undermine reasonable uses of health care data, it does not offer an immediate answer to information reuse dilemmas, nor does it alter the risks of re-identification in de-identified datasets. These legal protections need both understanding and interpretation before uses of information can be governed in practice.

### Requirements and Motivations: Risk Management in Practice

The legal requirements must nevertheless be enacted in practice. Data Protection Act principle 7 requires data to be handled securely; however, enacting this requirement in practice is not a simple or trivial task. Perhaps the most authoritative resource for developing information security management is the ISO 27000 series of standards [47]. Within this series the most pertinent standards are 27001 (which defines the requirements for information security) [48] and 27002 (which defines a code of practice for implementation of the elements of ISO 27001) [49]. An accredited ISO auditor can certify compliance with 27001 independently, while 27002 relies on an understanding of and success criteria set by the organization that is implementing the requirements established in 27001. This makes it difficult to certify independently, but it is certainly internally auditable. A prime example of ISO 27002 exists in the form of the Information Governance Toolkits and their variations across UK jurisdictions [50]. These have been developed to incorporate requirements from legislation and good practice guidelines for organizations that handle health care information and provide a basis for establishing levels of compliance.

A key element of 27001 and its certification is to define the scope of the security requirements. It then mandates the development of an information security management system (ISMS), which must be well supported by management and responsible parties. The ISMS provides a basis for organizations to run risk assessments and analyses on data use, and to refine the findings into mitigation strategies that are developed in policies for data use. These policies must be understood by the people that they are supposed to govern and must define a basis for configuration of software tools responsible for access control and privilege management. There is a focus on engagement for and with people working with information, which in turn mandates that they should be well informed and guided in working practice. Bearing in mind the particulars of security practicalities, the safe haven concept is focused on mitigating risks, whether risks to participants and their re-identification, risks to organizations who process the data, risks to organizations who have control and responsibility for the data, or risks to continuing research and public appetite for the support of research.

To summarize, ISO 27001 allows for an independently certifiable process to show that organizations are compliant with the internationally recognized core requirements of good information security practice, while ISO 27002 provides a basis to contextualize those core requirements through the Information Governance Toolkits in the context of health care research. Recognizing these criteria, the apparent evolution of the safe haven concept has included work in the research community to seek independent certification for compliance with ISO 27001 to provide additional practical security and support for research communities as well as public reassurance. While these help provide assurance that some of the 12 criteria provided by Burton et al [4] are met, the extent to which this reassurance supports trustworthiness remains unclear.

### Requirements and Motivations in Context: Evolution of the DSH Paradigm Through Information Governance Research

The 2013 second Caldicott review of information governance recognized that the research community had worked hard to overcome perceived impediments of information governance when handling health care information for purposes beyond health care, that “significant lessons regarding data sharing from public health and research” and “...the approach to information governance adopted in public health and research may be helpful...” to other sectors [36]. The next section focuses on the experience of what this means in practice using 4 independently developed examples of DSHs across the United Kingdom to illustrate the practicalities and the need to involve and engage with the wider public to satisfy their interest in research work and understand their concerns over the use of health and social care records.

We discuss the examples of the 4 nodes of the Farr Institute of Health Informatics Research as small case studies to illustrate the developing paradigm. The Farr Institute comprises 4 nodes across the United Kingdom: one in Wales, one in Scotland, one in the southeast of England, and one in the north of England.

The Institute was founded in 2013 and has incorporated a series of research platforms that have been developed independently of each other in partnership with research funders and local NHS trusts and health boards. Each of these nodes has also developed and evolved its own information governance frameworks, systems, and processes. The Welsh and Scottish examples have achieved international recognition for their initiatives [51], and the English examples have achieved independent ISO certification in line with the recommendations in the second Caldicott review. But do these examples represent a common view of the original safe haven concept? We discuss the 4 nodes in the next sections, which are structured according to the common features identified across each node that have emerged during the case studies.

## Safe Havens in Research: Farr Institute Node Case Study Examples

### Farr Health eResearch Centre (North England)

#### Core Governance Framework

The Farr Institute Health eResearch Centre in north England is a collaboration between 4 universities in the region, the NHS, and industry. It is governed by a steering group that meets periodically to develop and maintain strategy, as well as to monitor performance of the Centre and its facilities. This steering committee comprises senior representatives of the universities involved with the Centre (including Liverpool, Lancaster, and York), independent NHS representatives, users, and industrial collaborators, as well as patients and members of the public.

#### Independent Ethical Review, Certification, and User Accreditation

The Centre will host a DSH at the University of Manchester, where the equipment on which it is run is held within a physically secure environment. This includes the infrastructure for data storage, archiving, and networking that serves academic research collaborators and includes connections to components held within the NHS network. The safe haven is compliant with the requirements of an ISO 27001 ISMS, where some components have achieved independent certification and the others are expected to have done so by early 2017. The NHS networked component is compliant to level 2 of the Information Governance Toolkit and is run within the governance framework of the NHS. The safe haven and its use are governed by security policies and standard operating procedures in line with the ISO ISMS. Once projects have received required approved, the safe haven provides both NHS users and researchers with secure local and remote access to virtual machines that offer a suite of analytics tools tailored to the analysis needs of their projects.

#### Cataloguing and Data Management

This suite of tools, termed the dLab (for data laboratory), will provide researchers with a dataset catalogue, providing metadata descriptions of data available within the safe haven environment. The dLab will further provide desktop access to data, applications, compute power, and storage, along with appropriate authentication, authorization, and auditing infrastructure. The safe haven offers additional features to link datasets where appropriate permission has been granted and an archiving feature for virtual machines on which analyses have been run once the researchers have confirmed they are completed. Additionally, an eLab data management facility [52] will be provided to researchers. Where appropriate to the level of sensitivity of data being accessed, both the dLab and eLab components of the safe haven will provide remote desktop access using 2-factor authentication. In the longer term, the dLab software stack will be provided to the equivalents in the other Farr Institute partners for exchange of scripts, data, and research objects [53], with the potential for implementing a single sign-on mechanism between Farr Institute partners. The implementation of remote access is designed to reduce the need for additional copying and physical transfer of data. Additional facilities within the safe haven include a data deposit facility to receive sensitive datasets on behalf of Farr Institute Health eResearch Centre consortium members. Pseudonymized data can be received from NHS partners through periodic data feeds via the N3 network, again mitigating any need for excess copying or physical transportation of data.

#### Future Ambitions and Developing Protection: Opportunities for Public Involvement

In addition to existing approvals requirements, the Centre is working toward establishing an independent governance board, comprising both expert and lay members, to review research project proposals and approve them before the researchers can have access to the tools and datasets that they need to answer their research questions. The Centre intends to make any approvals dependent on the governance board’s assessment of the scientific validity of the project’s proposed research questions in combination with the results of independent ethics reviews. The governance board will also approve the researchers themselves, and this relies on ensuring the researchers have undertaken information governance training as required by the standard operating procedures.

### Farr Centre for Improvement in Population Health through E-records Research (Wales)/Secure Anonymised Information Linkage Databank

#### Governance Framework

The Centre for Improvement in Population Health through E-records Research (CIPHER) (Wales) node of the Farr Institute uses the Secure Anonymised Information Linkage (SAIL) Databank at Swansea University. Conceptualized in 2006, SAIL has since been evolving continually. At the heart of the SAIL model was and is the need to find and maintain a balance between preserving individual-level privacy and harnessing the potential to use health-related data to their full potential for the benefit of public health [54]. Seven essential objectives were set: secure data transportation, reliable data matching between datasets, robust anonymization and encryption, disclosure control, data access controls, scrutiny of data utilization proposals, and external verification of compliance with information governance. SAIL has developed in partnership with NHS Wales and continual consultation with the Welsh Government, regulatory bodies, and professional and public groups.

#### Independent Ethical Review, Certification, and User Accreditation: Opportunities for Public Involvement

SAIL insists on data sharing agreements being in place between SAIL and all data providers. Through the SAIL gateway, data are provided to each project on a predetermined basis. All research proposals are submitted to an independent information governance review panel, which includes representation from the British Medical Association, Public Health Wales, NHS Wales Informatics Service (NWIS), National Research Ethics Committee, and the public (members of the Consumer Panel for Data Linkage Research). Approval is given only if the research is appropriate and in the public interest, and the research can proceed only on receipt of full approval from this panel. Project analysts are then assigned permissions within the SAIL gateway to match the independent information governance review panel application, with access controlled through an automated security system. Project-specific data views are created to provide tailored data subsets.

All persons accessing the SAIL gateway have to be approved researchers (have undergone accredited training) and are required to sign a comprehensive data access agreement about their use of the data in SAIL. The research is carried out within the SAIL secure gateway environment. Results can be taken out only via a request process, which involves scrutiny by SAIL senior analysts for information governance issues, such as small cell counts, and other breaches of the SAIL output release policy.

Access to the SAIL databank is remote, via a firewalled virtual private network known as the SAIL gateway. It uses enhanced user authentication, auditing of all SQL commands, and configuration controls to ensure that data cannot be removed or transferred unless authorized.

#### Cataloguing and Data Management

Robust anonymization is provided by a trusted third party, NWIS. All data are transferred using Web-based secure file upload facilities, with incoming datasets being split into a demographic component (personally identifiable information) and a clinical or event component. The demographic component is sent to NWIS, which then assigns an anonymous linking field to each individual, thus ensuring anonymity and encryption. The clinical component is sent to SAIL. At SAIL, the anonymous linking field is linked to the clinical or event data and reencrypted.

#### Future Ambitions and Developing Protection

SAIL is engaged in a constant program of improvement and has moved to a purpose-built data science building, which will also house the Administrative Data Research Network. The physical security for the new data science building will be configured such that it will accommodate successfully the physical security requirements for all projects and research programs based within the building, including the storage of Administrative Data Research Centre for Wales de-identified government data (classified to official/official sensitive) requiring the highest level of security (security zone 5) within the building. The external ISO 27001:2013 ISMS certification process for the SAIL program was completed in November 2015.

### Farr Scotland/Scottish Health Informatics Programme

#### Governance Framework

The Scottish node of the Farr Institute builds on the progress and success of the Scottish Health Informatics Programme (SHIP), which ran from 2009–2013. Through SHIP, a principled proportionate governance model was developed in order to streamline research applications and approvals for data linkage, while simultaneously ensuring that research was scientifically sound and ethically robust. Risk mitigation played a central role within the SHIP model, and access to health data for research was contingent on performing a privacy risk assessment and meeting the benchmarks of safe people, safe environments, and safe data, as described by Sethi and Laurie [55]. Farr Scotland [56] is building on these contributions (and requirements) from SHIP in tandem with the Scotland-wide Data Linkage Framework, the Scottish Informatics Linkage Collaboration, National Records of Scotland’s Registrar General, and the Administrative Data Research Centre.

#### Independent Ethical Review, Certification, and User Accreditation: Cataloguing and Data Management

Access to the national safe haven and national data (located at the NHS National Services Scotland) is provided via the electronic Data Research and Innovation Service. This service assigns (approved) researchers (who have undergone accredited training) to a dedicated research coordinator who offers support for the process of submission of the initial data access application (including study design and coding) right through to data analysis. All data uses must abide by the key benchmarks set out under SHIP. The research coordinator also acts as an intermediary between data controllers and researchers, who must all abide by the Guiding Principles for Data Linkage established by the Scottish Government. Streamlined approval for access to more than one NHS board dataset for research purposes was granted by the Privacy Advisory Committee for Scotland which, as of May 1, 2015, is to be subsumed under the new Public Benefit and Privacy Panel for Health and Social Care.

The Scottish Government is leading the establishment of procedures to provide independent accreditation of safe havens (safe settings), mechanisms for monitoring compliance (safe projects), guidance on coding, terminology, and disclosure (safe outputs), and the development of training for researchers (safe people). A significant challenge for the Farr Institute is that Scotland lacks legislation “defining the status of accredited safe havens, but the review of the Patients’ Rights Act, due in 2016, may provide an opportunity to make clear in law the status of the safe havens” [57].

#### Future Ambitions and Developing Protection: Opportunities for Public Involvement

The Farr Institute will be embedded within a network of safe havens, which includes the NHS National Services Scotland national safe haven and 4 lead NHS Research Scotland nodes. Quite what this network will look like and how it will operate is still very much under development. The national safe haven currently consists of 2 stand-alone computer terminals that accredited researchers can access remotely via a secure network or server.

The recent Scottish Government report *A Health and Biomedical Informatics Research Strategy for Scotland* [58] considers the potential and challenges involved with establishing such a network of safe havens. It has identified the following key challenges in order to facilitate interoperability between safe havens: technical challenges, the practical details of how a network of safe havens should operate, and determining whether a single point of entry should be necessitated (or whether there can be multiple points of entry). On this latter issue, a balance must be achieved between having a single point of entry, and support and provision of local expertise for researchers. Indeed, additional safe havens may be established, and the question arises as to whether these safe havens can join the network and, if so, which standards and accreditation procedures they will be subject to. In this vein, a Safe Haven Charter for Scotland (based on the core principles of ISO 27001) is being developed, which will include a set of high-level principles around technical, practical, and overarching governance considerations [59]. The biggest challenge will be striking a further balance between determining and meeting common and consistent data standards while facilitating flexibility between local nodes. Farr Scotland has a dedicated work stream committed to civic engagement and will strive to explore and feed in to governance approaches and public attitudes around such uses of data.

### Farr London

#### Core Governance Framework

The London node of the Farr Institute is a collaboration between University College London, the London School of Hygiene & Tropical Medicine, and Queen Mary University of London. The DSH has been established within the School of Life and Medical Sciences at University College London as an identifiable data handling service, comprising a technical solution for the secure storage of identifying or pseudonymized data, and a service within which the technical solution is mapped that provides individual health research projects guidance on how to develop their own working practices and achieve Information Governance Toolkit compliance.

#### Independent Ethical Review, Certification, and User Accreditation

The research projects running within the Farr London node are subject to their own contractual obligations with data providers, as well as independent ethical approvals and oversight, where any changes to approved information handling, linkage, or wider sharing must be authorized by the ethics committee that provided the original approvals via University College London, the London School of Hygiene & Tropical Medicine, or Queen Mary University of London boards, or the NHS research ethics committees, where needed.

The technical solution comprises a “walled garden” approach, which uses secured virtual sessions run from within a secure infrastructure. This element has achieved ISO 27001:2013 certification and is audited annually by accredited ISO auditors. All steps use a 2-factor authentication, and the session forbids any download of data (including copying and pasting and some screen capture). All projects are logically segregated from each other within the safe haven, and access is controlled and permitted only to those users who have been registered and attended information governance awareness training courses, as well as completed online information governance tests annually for their reaccreditation.

The identifiable data handling service provides guidance on how to achieve appropriate levels of Information Governance Toolkit compliance, preparation for seeking Section 251 exemption from the common law duty of confidentiality where applicable, and wider information security framework development, including the drafting and execution of data sharing agreements and codes of practice. The identifiable data handling service also routinely tours the partner institutions with awareness sessions and runs training courses and the online annual information governance reaccreditation tests for registered users. In addition to this, the identifiable data handling service is governed by a user group, which routinely meets and offers usage feedback to the School of Life and Medical Sciences, and an executive project board, which oversees budgeting and approves the execution of upgrades and changes to the service and systems. The outreach to the user community is tailored to help them understand the security and good practice requirements and the change in working behavior within this managed environment.

#### Cataloguing and Data Management

The technical solution also includes a patient indexing service, which is based on bespoke de-identification and record linkage software developed by Belgian security company Custodix [60]. This service allows for datasets to be anonymized or pseudonymized where appropriate, so that these datasets can be securely shared under any required authorization with other Farr Institute nodes or authorized research collaborators. The linkage software can merge records across different projects held within the safe haven where this is permissible. Functionality includes a feature where clinical data sources are, on registration, able to upload identifiable datasets securely using a dedicated upload service. Research project recipients are then able to access the uploaded data and transfer it to a suite of licensed database and analytical tools over a secure virtual session.

#### Future Ambitions and Developing Protection: Opportunities for Public Involvement

The identifiable data handling service is considering the establishment of an ethics oversight committee to include a panel of researchers, clinical and legal expertise, and involvement from patient groups or members of the public to help consider any ad hoc collaborations across research projects or wider interventions.

## Discussion

### A Common Paradigm?

Across the 4 Farr Institute nodes, common features of the information governance frameworks have been developed. In all cases, there is a recognized compliance with the Information Governance Toolkit or the Scottish equivalent. The English nodes have been certified to ISO 27001, and the CIPHER node received certification in November 2015. Each node comprises or is in the process of establishing a series of committees and panels for oversight, development, and governance, with some cases including public and lay representation. Each node also requires that researchers undertake training and education before they can use the facilities.

The following appear to be consistent features for a safe haven across the Farr partners that build upon the 12 criteria offered by Burton et al [4] and the need identified by Knoppers and Chadwick [5] for expanding the definition of trust to include the wider public and their trust in security:

1. Independent certification for establishing good working practice, which includes a focus on people and behaviors when handling information and the development of steering committees and working groups

2. Training, education, and accreditation of people who work within the environment, including assessment and professional certification

3. Working practice within the prescription of jurisdictional legislative relief, which includes reviews by ethics committees for research activities

4. Cataloguing and data management, which includes an updated resource for defining not only what data are available, but also the requirements for using them in research within these environments

5. Participant contact for research or appropriate exemptions under the law

6. Developments in protection and future ambitions

7. Opportunities for public engagement and involvement, including events and workshops to disseminate research findings, as well as having lay representation on panels, steering committees, and working groups. This helps ensure that the public have a voice in the policy, use, and development of the infrastructure.

### Is This Enough?

Our proposed common definition illustrates the key aspects for developing the DSH paradigm into trusted platforms for clinical research. It emphasizes that we must implement and maintain concrete examples of what is safe in terms of protecting participants and researchers, and what is trusted by those same participants, funders, the academic research community, and the wider public. This common definition builds on the criteria established by Burton et al [4] and takes into account the need for a more inclusive understanding of what is meant by trust, reinforcing the proposals of Knoppers and Chadwick [5]. This work further develops these themes and findings by providing not only exemplars of how these aspects are established in practice, but also a proposed framework for the ongoing evolution away from the static notion of the safe haven as a physical environment alone. It is moving the understanding toward a trusted research platform that handles societal, individual, and professional concerns, and offers reassurance and the opportunity to govern its operation beyond the research and regulatory communities. It supports the notion that an environment view must also include the people who work in, govern, and contribute to that environment, and their support. Trust must be won and nurtured, and it will vary according to the stakeholders who are involved in doing research, or indeed about whom the data have been collected; this relies on involvement and informed dialogue.

Such a requirement will not be met by focusing on the integrity, reliability, or security of the technical solutions within the platforms themselves in isolation from the training needs of the researchers and their education of what good working practice entails. Nor can this in turn be handled in isolation from independent ethical oversight of how data can be used, or without encouraging and supporting lay representation on steering groups for the platforms or research consortia that use them. The provenance of the data themselves must provide assurance to the research community that the data are fit for the purposes of their research, but cannot be the focus of efforts without ensuring that they are adequately catalogued. Critically, none of these aspects can be isolated from ongoing public engagement and education, which involves a 2-way communication between the academic research community and the public about how information is used and what the benefits are.

To fully articulate what we mean by safe and trusted, we must reemphasize that at the core of the DSH paradigm is the notion of risk management. We have discussed how risks of participant identification remain regardless of the methods used to render records anonymous, and we have highlighted that the research community needs more identifiable attributes for realistic utility and should not handle risk management across datasets in isolation, at the cost of reasonable use and sharing. The DSH paradigm is ultimately about managing those risks, so no basis for an open dialogue with the public or their meaningful involvement can take place without being transparent about the existence of those risks. But the DSH approach does not guarantee, and nor should it, that risks will not remain; rather, they operate within an independently certified environment that will more likely be able to adapt to the changing nature of known and emerging risks, with due respect to interest from the public and their concerns, and ongoing mindfulness of the ethics around the research, its data use, and its outputs. Such environments are made up as much of people and their actions as of hardware, software, and policies.

It is for individual members of the public to decide how they feel about the ways in which information recorded about them is being looked after, and while they do not always get a say in whether information is shared for purposes other than their direct care, the DSH paradigm must emphasize the importance of highlighting the benefits of the information sharing in spite of the risks of re-identification, at the very least to give people an opportunity to develop an informed opinion, rather than erroneously guaranteeing them a risk-free solution. To win the trust of any stakeholder, this means that we must encourage shared ownership of the problem with the public and patient communities while being transparent and open about how health information is used and why it is important that it is being used.

### Conclusions

We have described the motivations behind developing the DSH paradigm to support the big data, epidemiological research drive. In doing so, we have discussed the basis for the paradigm and introduced a series of requirements from a legal, ethical, and information security perspective, building on established work in this area. We have emphasized that these alone do not represent clear public anxieties about and interest in how research is conducted and information is protected. Through this discussion, we have proposed a common definition of the DSH paradigm by considering and describing the technical infrastructure, ethical oversight, researcher training and education process, the internal governance, and external, independent audit and public engagement and involvement drives of 4 independently established clinical research platforms and the common features among them.

We have critically reviewed the proposed definition by emphasizing the importance of involving the public and engaging with them openly and transparently, especially with regard to risks or re-identification and how the risks are managed. The focus of the DSH paradigm cannot be solely on technical or procedural approaches to risk mitigation. Engagement with people is paramount, and not exclusively with the public but also the researchers who use the platforms underpinned by the DSH paradigm. This includes responding to their educational needs and supporting their ability to do the research with guidance on ethical requirements and due diligence for understanding funder requirements. It is particularly vital to understand the needs and expectations of all these stakeholders if the clinical research community is to inspire trust in their research platforms. While this paper has focused on experiences across the United Kingdom, the findings will be of interest internationally to help manage the challenges that exist for electronic health records reuse in clinical research.

## Abbreviations

CIPHER: Centre for Improvement in Population Health through E-records Research
DSH: data safe haven
ISMS: information security management system
ISO: International Organization for Standardization
NHS: National Health Service
NWIS: NHS Wales Informatics Service
SAIL: Secure Anonymised Information Linkage
SHIP: Scottish Health Informatics Programme
